# Personal and Selfish

**Published:** 1866-11

**Authors:** 


### PERSONAL AND SELFISH.

A NUMBER of years ago I was impelled to abandon the general practice of medicine and surgery, on account of physical unfitness for the discharge of its duties. Not willing to adopt a course of life that would render my knowledge of medical science unavailing, I turned my attention to the specialty of Dental Surgery, with what success is known to the profession. Unfitted again, for prolonged operations at the chair, it has become necessary either to abandon medical practice altogether, or to adopt another modification. Unwilling to forsake the favorite science of my youthful days, I resolved on the following course:

I have opened an office for the treatment of all the diseases pertaining to or resulting from the derangements of dentition and the dental organs, but do not propose to fill teeth or insert artificial work. Diseases of the antrum, facial neuralgia, chronic alveolar abscess, absorption of the alveolar processes, diseases of the gums, etc., are often an annoyance to either the physician or the dentist, while their proper treatment implies a knowledge of both professions. I also propose, the advice of others corroborating, to use the nitrous oxyd in extracting teeth, and in any operations that require it. In short, the aim will be to make myself useful to both the profession and the public. The use of anæsthetics in ordinary dental offices is attended with so much inconvenience as to render it almost impracticable. When it is made a special business the case is different. No one will question that any thing which takes a patient so far toward the spirit world that he loses consciousness of this one, ought to be in careful, experienced, and educated hands. Hoping to have still the confidence and coöperation of the readers of the REGISTER in my new experiment, I am as ever,

